# Novel Approaches for Estimating Female Sex Worker Population Size in Conflict-Affected South Sudan

**DOI:** 10.2196/11576

**Published:** 2019-03-18

**Authors:** Alfred Geoffrey Okiria, Alex Bolo, Victoria Achut, Golda Ceasar Arkangelo, Acaga Taban Ismail Michael, Joel Sua Katoro, Jennifer Wesson, Steve Gutreuter, Lee Hundley, Avi Hakim

**Affiliations:** 1 IntraHealth International Juba South Sudan; 2 United States Centres for Disease Control and Prevention Juba South Sudan; 3 South Sudan Ministry of Health Juba South Sudan; 4 IntraHealth International Chapel Hill, NC United States; 5 United States Centres for Disease Control and Prevention Atlanta, GA United States

**Keywords:** sex workers, population, methods, South Sudan

## Abstract

**Background:**

Limited data exist describing the population size of female sex workers (FSW) in South Sudan. A population size estimation exercise among FSW was undertaken in Juba and Nimule during the Eagle Survey.

**Objective:**

The study aimed to estimate the number of FSW in Juba and Nimule to inform resource allocation and service provision for FSW.

**Methods:**

We utilized service and unique object multipliers, and 3-source capture-recapture methods in conjunction with a respondent-driven sampling (RDS) survey to estimate the number of FSW in Juba and Nimule. For service multiplier, the number of FSW testing for HIV in 2015 (Juba) and 2016 (Nimule) was obtained from the LINKAGES program targeting FSW. Survey participants were asked whether they had been tested for HIV by LINKAGES during the relevant period. A total of 2 separate unique object distributions were conducted in Juba and Nimule. In Nimule, these were combined to produce a 3-source capture-recapture estimate. The exercise involved distribution of key chains and bangles to FSW, documentation of the number of those who received unique objects, and questions during RDS survey to assess whether participants received unique objects.

**Results:**

In Juba, the service multiplier method yielded an estimate of 5800 (95% CI 4927-6673) FSW. The unique object estimate (key chain and RDS participation) yielded 5306 (95% CI 4673-5939). Another estimate using RDS participation and receipt of a bangle yielded a much lower estimate of 1863 (95% CI 1776-1951), as did a 2-source estimate of key chain and bangle (2120, 95% CI 2028-2211). A 3-source capture-recapture estimate could not be produced because aggregate rather than individual level data were collected during the third capture. The multiplier estimate using key chain and RDS participation was taken as the final population estimate for FSW in Juba, which constitutes more than 6% of the female population aged 15 to 64 years.

In Nimule, the service multiplier method yielded an estimate of 9384 (95% CI 8511-10,257). The 2-source estimates for key chain and RDS yielded 6973 (95% CI 4759-9186); bangles and RDS yielded a higher estimate of 13,104 (95% CI 7101-19,106); key chains and bangles yielded a lower estimate of 1322 (95% CI 1223-1420). The 3-source capture-recapture method using Bayesian nonparametric latent-class model-based estimate yielded a population of 2694 (95% CI 1689-6945), and this was selected as the final estimate for Nimule, which constitutes nearly 40% of female population aged 15 to 64 years.

**Conclusions:**

The service and unique object multiplier, and 3-source capture-recapture methods were successfully used to estimate the number of FSW in Nimule, whereas service and unique object multiplier methods were successfully used in Juba. These methods yielded higher than previously estimated FSW population sizes. These estimates will inform resource allocation and advocacy efforts to support services for FSW.

## Introduction

### Background

South Sudan, the newest country in the world, gained independence in 2011. This was followed by the return of refugees and foreigners from neighboring countries. The HIV prevalence in the neighboring countries is 6% in Uganda [1] and 5% in Kenya [2], and it is higher than 2.4% in South Sudan [3]. The relative stability allowed increased commerce and the apparent increase in the number of female sex workers (FSW) [4]. Little data exist on sex workers in South Sudan. Programs for FSW are guided by mapping conducted in 2011 and 2012 and formative assessments conducted in 2014. Despite a period of relative calm after independence, South Sudan experienced political crises in December 2013 and July 2016 that resulted in significant population movements [5].

Previous mappings and formative assessments indicated that Juba was home to the largest number of FSW in the country. The South Sudan AIDS Commission and World Health Organization mapped a study in 2012, which estimated 2511 (range: 2013-3008) FSW in Juba and 378 (range: 316-439) in Yambio [4,6]. Nimule, on the South Sudan-Uganda border, was estimated to have 400 sex workers [7].

Sex work is illegal in South Sudan and sex workers operate in a very stigmatizing environment with the constant threat of arrest, imprisonment, and sexual exploitation [6,8]. This context has led many sex workers to operate in a covert manner, in lodges, brothels, and homes. Street-based sex work is less common [4,6]. Few health services targeting FSW exist in South Sudan and until recently the South Sudan Ministry of Health (MOH) had limited data describing sex workers in the country to inform service provision.

Accurate estimation of the size of key populations using empirical methods that include data collection rather than conjecture provides important data for advocacy and resource allocation of HIV programs [9]. These estimates further facilitate intervention planning, monitoring and evaluation, and epidemic modeling. Reliable estimates of the size of key populations who are hidden, outlawed, stigmatized, and highly mobile are difficult to obtain [10]. There is no gold standard for size estimation, which sometimes prompts researchers to use multiple methods to identify a single best estimate [9,11,12]. Common size estimation methods include mapping and census, both of which provide underestimates of key populations, successive sampling, multiplier method, and capture-recapture [13-15]. The latter method was originally developed to count wildlife populations [16]. The method has evolved to include significant improvement in the accuracy and reliability of estimates, particularly through the use of 3 or more sources for capture-recapture, and the method may produce the best estimates available [9].

### Objectives

The objective of this activity was to estimate the number of FSW in Juba and Nimule to inform resource allocation and service provision for FSW. This was done by the MOH together with the US Centers for Disease Control and Prevention (CDC) and IntraHealth International, who together implemented the first biobehavioral survey (BBS) and empirical size estimation activities, known as the Eagle Survey, among FSW in South Sudan.

## Methods

### Study Population

We included, in the survey and related size estimation activities, women and girls aged 15 years or more who received money, goods, or services in exchange for sex in the past 6 months; spoke English, Juba Arabic, or Kiswahili, and lived or worked in Juba or Nimule. Sexually exploited children and adult survivors of violence were referred to partner organizations experienced in providing counseling, health, social, and other protective services to these populations.

### Procedures

The Eagle Survey utilized the service multiplier, unique object multiplier, and 3-source capture-recapture method to estimate population size of FSW. For the service multiplier, the number of FSW testing for HIV in Juba from January to December 2015 and from January to December 2016 in Nimule was obtained from the LINKAGES program that targets FSW. RDS survey participants were asked in an interview whether they had been tested for HIV by LINKAGES during the relevant period. All FSW getting services from LINGKAGES receive unique identification numbers that are used each time they access services. This ensures that data from LINGKAGES are unique counts reflecting the number of people tested rather than the number of tests conducted.

The second method used in both Juba and Nimule was the unique object multiplier method. This involved the distribution of key chains and bangles as unique objects to FSW. Paired together with the RDS survey, these 2 separate object distributions were used to produce a 3-source capture-recapture estimate.

Data collection began in Juba with the distribution of unique key chains to FSW in October 2015. After 5 weeks, data collection for the RDS survey began. Finally, in June 2016, unique bangles were distributed to FSW. In Nimule, unique key chains were distributed in June 2016 followed by a distribution of bangles 1 week later. The RDS survey was meant to begin 1 week after the bangle distribution but an eruption of violence days after the bangle distribution led to the postponement of the survey until January 2017. It was not possible to distribute new unique objects at that point.

In both Juba and Nimule, the number of FSW receiving a key chain during the distribution was the first data source. For Juba, the second data source was the RDS survey, which included questions to assess whether the participant had received a key chain. The third data source was the number of FSW who received a bangle after the RDS survey. Lessons learned in Juba resulted in a change in the sequence for Nimule with the unique object distributions occurring before the RDS survey.

Unique object distribution was conducted by FSW volunteers identified largely through LINKAGES. They were of different nationalities, operating in different parts of the city, and they were literate, respected, influential, and had the ability to explain the purpose of the object to potential recipients. In Juba, 54 FSW distributed key chains and 30 FSW distributed bangles, 6 of whom distributed both. In Nimule, 22 FSW distributed key chains and 14 FSW distributed bangles, 4 of whom distributed both. A 1-day volunteer training was conducted before each distribution covering the following: study background and objectives, eligibility criteria, safety and security, how to approach FSW, how to complete the distribution registration sheet and the unique object distribution form, the distribution process, and role plays. A second day was added for the key chain distribution training in Juba given the large number of volunteers. To facilitate recall and identification of volunteers, all volunteers wore a blue T-shirt bearing an eagle logo (the survey symbol). Volunteers were instructed to distribute objects to FSW in their neighborhoods.

In Juba, approximately 5 weeks before the start of the RDS survey in November 2015, the survey staff and volunteers identified and mapped hot spots with support from the LINKAGES project and with the aim of distributing at least 1300 key chains to FSW. Each of the FSW encountered received only 1 key chain, which she was instructed to keep because she may be asked about it in the near future by the survey staff. Volunteers used registration sheets to keep track of when, where, and how many objects were distributed and whether individuals had already received or rejected the object. Objects that were not distributed were returned to the study coordinator. Volunteers also told FSW about the Eagle Survey and let them know that they would not face stigma from the survey team. The volunteers received compensation for their time and transport to distribution locations.

Upon receipt of the object, the FSW was considered captured. Volunteers tracked the number of objects they distributed as this indicated the number of participants captured. During the RDS survey’s eligibility screening process, all FSW were asked by the coupon manager whether they received the unique object, to show or describe it, and how they received it. Those who could not show or describe the object were asked to identify the correct object from a panel of 5 similar objects. The third recapture in Juba was a bangle distributed 3 months after the end of the RDS data collection because of procurement challenges. It utilized the same methods as the first unique object distribution. During the bangle distribution, FSW were asked if they received a key chain, participated in the RDS survey, and received a bangle.

In Nimule, the order of activities was slightly modified. The 564 key chains were distributed in June 2016 followed by 546 bangles distributed 2 weeks later. Due to the conflict that started in July 2016, the RDS survey was delayed till January 2017. During the bangle distribution, participants were asked if they received a key chain or a bangle. The RDS survey again asked if the person received a key chain, bangle, or both. In both locations, all objects were distributed within 1 day. The number of key chains and bangles distributed was estimated to cover the entire FSW sample, 910 for Juba and 400 in Nimule with 40% extra key chains/bangles.

### Analysis

The 2-source multipliers were calculated using the standard formula described elsewhere in conjunction with weighted estimates from the RDS survey or nonweighted estimates in the case of the key chain-bangle estimates [9]. The 3-source capture-recapture estimate was calculated using a Bayesian nonparametric latent-class capture-recapture model as implemented in the R package [17].

### Ethical Approval

The study protocol was approved by the Government of South Sudan MOH Research Ethics Committee and CDC's Center for Global Health Associate Director for Science.

## Results

### Main Findings

**Figure 1:**
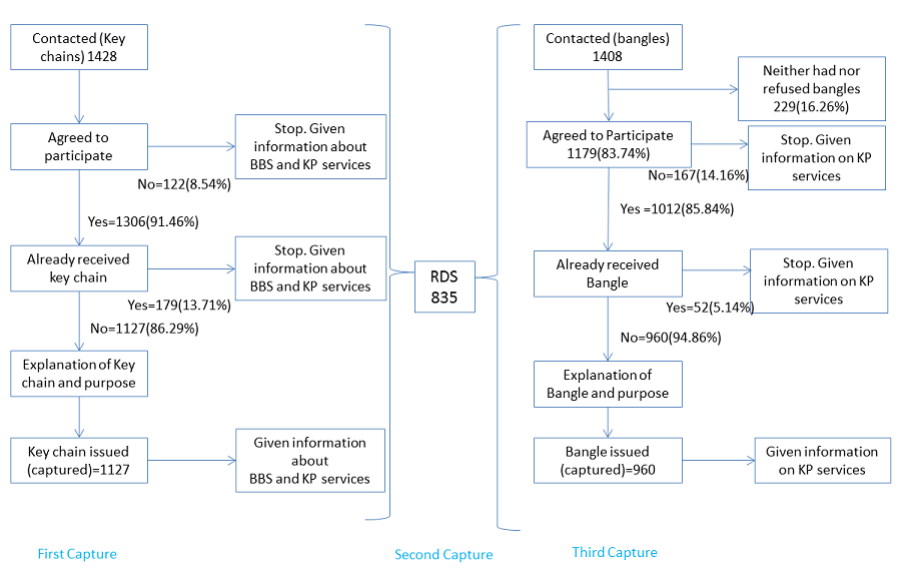
Population size estimation process in Juba. BBS: biobehavioral survey; RDS: respondent-driven sampling; KP: key population.

In Juba, volunteers distributing key chains contacted 1428 FSW across 10 neighborhoods (Figure 1). Approximately 9 in 10, that is, 86.29% (1127/1306) of the FSW were not in possession of a key chain agreed to receive it. About 13.71% (179/ 1306) of the FSW contacted had already received a key chain. During the RDS survey, 179 FSW reported receiving a key chain. More neighborhoods where women sell sex were identified during the RDS survey; consequently, volunteers distributed bangles in 28 neighborhoods. Volunteers distributing bangles contacted 1179 FSW. Similar to the key chain distribution, 85.84% (1012/ 1179) of the FSW contacted had agreed to participate. Of those that participated, 94.86% (960/1012) of the FSW agreed to receive a bangle whereas 5.14% (52/1012) of the FSW had already received a bangle.

In Juba, LINKAGES service data indicated that 2204 FSW tested for HIV in 2015. The RDS survey included 835 FSW participants, 323 of whom tested for HIV at LINKAGES-Juba in 2015. Of the 9 seeds in the RDS survey, 3 were affiliated with LINKAGES. The longest chain had 17 waves. Among RDS participants, 314 received a key chain and 33 presented it during eligibility screening. A total of 498 RDS participants received a bangle.

The service multiplier method yielded an estimate of 5800 (95% CI 4927-6673) FSW. The unique object multiplier method of key chain and RDS participation yielded a similar result of 5306 (95% CI 4673-5939) but with tighter uncertainty bounds. Another unique object multiplier estimate using RDS participation and receipt of a bangle yielded a much lower estimate of 1863 (95% CI 1776-1951), as did an estimate produced by key chain and bangle (2120, 95% CI 2028-2211), as represented in Table 1. A 3-source capture-recapture estimate could not be produced because aggregate rather than individual level data were collected during the third capture. We should have asked a few more questions to be able to better disaggregate the data to know the precise number of FSW who were in each capture. The setup of Juba forms would not allow for this.

**Figure 2:**
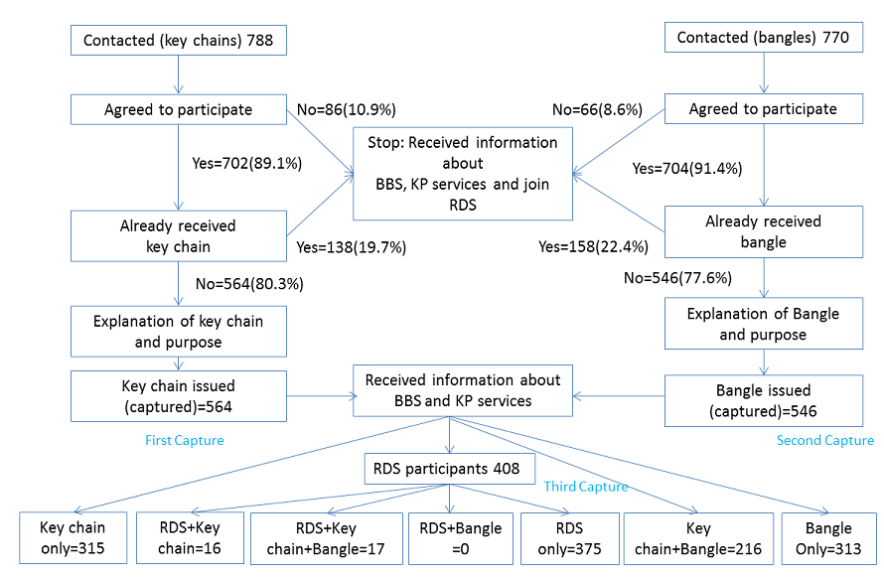
Population size estimation process in Nimule. BBS: biobehavioral survey; RDS: respondent-driven sampling; KP: key population.

In Nimule key chain distribution, 22 volunteers contacted 788 FSW in 20 neighborhoods (Figure 2). Approximately 9 in 10, that is, 89.1% (702/788) of the FSW agreed to participate, of which 80.3% (564/702) accepted to receive a key chain. Nearly 1 in 5, that is, 19.7% (138/702) of the FSW had already received a key chain. Furthermore, 3 weeks after the key chain distribution, 14 volunteers contacted 770 FSW in 20 neighborhoods. Approximately 9 in 10, that is, 91.4% (704/770) of the FSW agreed to participate, of which 77.6% (546/704) accepted to receive the bangles. More than a fifth, that is, 22.4% (158/704) of the FSW had already received the bangles.

In Nimule, LINKAGES service data indicated that 2204 FSW tested for HIV in 2016. The RDS survey included 408 participants, out of which 31 tested for HIV at LINKAGES-Nimule in 2016. Of the 7 seeds in the RDS survey, 2 were affiliated with LINKAGES. The longest chain had 12 waves. Among RDS participants, 16 received a key chain, and 17 received a key chain and bangle. None received only a bangle. Of the 33 who received a key chain, 10 presented it during eligibility screening.

The service multiplier method yielded 9384 (95% CI 8511-10,257). The 2-source estimates for key chain and RDS yielded 6973 (95% CI 4759-9186); bangles and RDS yielded 13,104 (95% CI 7101-19,106), and key chains and bangles yielded 1322 (95% CI 1223-1420). The 3-source capture-recapture method using the Bayesian nonparametric latent-class model-based estimate yielded a population of 2694 (95% CI 1689-6945; see Table 1).

### Female Population Estimates

On the basis of the National Bureau of Statistics population estimate of 2015 for females aged 15 to 64 years for Juba city, the service multiplier method estimates that 6.79% (5800/85386) of the female population are FSW, and based on 2-source capture-recapture, considering the key chain and RDS, 6.21% (5306/85386) are FSW. Using the females aged 15 to 64 years population estimates for 2017 for Nimule and taking the 3-source capture-recapture population estimates; we determined that 39.68% (2694/6790) are FSW.

**Table 1:** Population size estimates for female sex workers in Juba and Nimule using service multipliers and capture-recapture methods.

City or town and method		Population estimate	95% CI	Proportion of females aged 15 to 64 years^a^
**Juba**		—^b^	—	85,386
	Service multiplier method^c^	5800	4927-6673	6.79
	Capture-recapture method^d^	—	—	—
	2-source (keychain and RDS^e^)	5306	4673-5939	6.21
	2-source (RDS and bangle)	1863	1776-1951	2.18
	2-source (keychain and bangle)	2120	2028-2211	2.48
**Nimule**		—	—	6790
	Service multiplier method	9384	8511-10,257	138.20
	Capture-recapture method	—	—	—
	2-source (keychain and RDS)	6973	4759-9186	102.70
	2-source (bangle and RDS)	13,104	7101-19,106	192.99
	2-source (keychain and bangle)	1322	1223-1420	19.47
	3-source (keychain, bangle, and RDS)	2694	1689-6945	39.68

^a^Indicates the National Bureau of Statistics population estimate of 2015 for females aged 15 to 64 years for Juba and estimates of 2017 for Nimule.

^b^Data captured under the separate source options.

^c^Service multiplier method: method used for population size estimation methods used in Juba and Nimule.

^d^Capture-recapture method: method used for population size estimation in Juba and Nimule using unique objects that resulted into the different coerced combinations as indicated in the table.

^e^RDS: respondent-driven sampling.

## Discussion

### Principal Findings

In Juba, the service multiplier and the key chain unique object multiplier yielded similar population size estimates. These estimates were higher than previous estimates developed through mapping [4]. This may be because the mapping exercise defined FSW as those who received money for sex, excluding those that received goods or services for sex. The mapping was only conducted at hot spots (venues) and may have excluded hidden subpopulations operating in homes and compounds that were reached through the unique object distribution and RDS. The BBS widely defined FSW as those who exchange sex for money or goods or services, and through the peer referral, it was able to reach the hidden subpopulations. We noted divergent population size estimates in Nimule across all methods. This may result from a violation of the assumption of a closed population, stemming from displacement of FSW and other people as a result of the 2016 conflict.

The service multiplier method relied on the LINKAGES program data. The LINKAGES program uses a unique identification code for those who are reached with HIV testing and counseling; however, the difficulty in correct identification of FSW during outreach community-level testing may have resulted in some members of the general population being included in the counts, increasing the number of FSW testing and therefore the LINKAGES service beneficiaries. Our service count in Juba included all of 2015 to ease participant recall; however, the RDS survey began in November 2015. It is highly unlikely that people who tested negative in the survey in November to December 2015 would have gone for testing again at LINKAGES before the end of 2015. Survey participants living with HIV would have had no need for HIV testing after participating in the survey. Therefore, we do not feel that using the LINKAGES data through the end of 2015 risked excluding survey participants to produce a sizeable impact on the size estimate.

The proportion of FSW in the female population aged 15 to 64 in Juba is close to the estimates of 5% obtained in Kenya [10,18]. However, the absolute number for population estimates for FSW varies across the East Africa region and elsewhere [10,19,20]. In Nimule, the female population aged 15 to 64 years, who are FSW, was higher than that found in East Africa and elsewhere [10,19,20]. This is a result of high estimates of absolute numbers for FSW in this cross-border town. Ferguson and Morris estimated the number of FSW along the northern transport corridor of East Africa that links Mombasa port in Kenya and the rest of East African countries, and they found a higher proportion of FSW compared with general population women in the cross-border town of Busia (between Kenya and Uganda) compared with similar size towns along the transport corridor that are not on a border [21]. Our findings from the BBS also indicate that many of the sex workers who sell sex in Nimule reside or work across the border in the town of Elegu, Uganda.

### Limitations

Capture-recapture has 4 conditions that need to be fulfilled to give reliable estimates [22]. First, encounter (capture) and recapture (second encounter) need to occur close to the capture visit (first encounter). The short time frame helps ensure that a very small number of sex workers move in or out of Juba and Nimule, meeting the condition of a closed population. However, this was not possible because of logistical challenges followed by operational challenges brought on by the conflict in the country. Second, all FSW should have the same probability of being captured. In order to address this, we mapped hot spots to facilitate volunteer selection. Unique object distribution occurred during the time FSW were most available. However, some FSW, particularly South Sudanese, operate in homes and likely had a lower probability of being captured. Third, we could not eliminate all dependency among samples; for instance, women captured during the first round may be more likely to be captured again if they recognize the volunteers or were recognized by volunteers. A positive dependency among samples, the most likely situation, will lead to an underestimation of the number of sex workers. The independence of samples is most important for multiplier and 2-source capture-recapture methods, but it can be relaxed for the 3-source method. The independence of service and unique object multiplier estimates was likely assured by using weighted estimates from the RDS survey. In addition, we used key chains and bangles that did not have significant monetary value but were unique and attractive so that they would be remembered by survey participants. Furthermore, volunteers wore distinct T-shirts to facilitate recognition and participant recall. Asking RDS participants to identify the key chain or bangle during the RDS survey helped ensure data quality and check that FSW were not biased to provide a certain response or guessing.

It was not possible to produce a 3-source capture-recapture estimate in Juba because we only collected aggregate data during the bangle distribution. Having finalized the RDS, we needed to have asked a few more questions during the distribution of the second unique object (bangle) to enable disaggregation of the data to precisely determine the number of FSW who were in each capture. The analysis method required individual level data at each capture, which were not collected. The 3-source would have given much more accurate results, though with wider confidence intervals compared with the 2-source.

The correct identification of sex workers at the hot spots is a key factor to the success of the object distributions. The use of FSW as volunteers was of utmost value [23]. Peers are not only in the best position to identify other FSW but they also build confidence and trust in potential participants. However, it is possible that that some FSW were missed because of nonidentification, especially those operating out of their homes. There also could have been recall bias given that the interval between object distribution and the start of the RDS survey was quite long in Nimule, as was the bangle distribution in Juba. Using the same volunteers for the distribution of both objects may decrease the independence of samples; however, given the limited number of such volunteers in our study, we do not think our results were impacted by this.

### Conclusions

By utilizing multiple size estimation methods that include people who are not easily counted in mapping activities, this study produced the robust estimates of the number of FSW in Juba and Nimule, South Sudan. Unsurprisingly, we found that the number of FSW in Juba and Nimule is higher than previously estimated. Despite the limitations brought about by the conflict in South Sudan, the 3-source capture-recapture method was successfully used to estimate the population of FSW in Nimule. It may also be the method best-suited for estimating the size of populations in conflict-affected settings and other environments with high mobility as the assumptions of independent samples are more relaxed and can be done without relying upon a BBS that can take months to plan and implement. These estimates will help inform resource allocation and advocacy efforts to support services for FSW in South Sudan.

### Recommendations

The implementing partners that provide services to the FSW should improve on data quality during collection by ensuring correct identification of FSW and deduplication of the records to facilitate the use of the service multiplier method. For the unique object multiplier and capture-recapture methods, we recommend having different volunteers distribute objects for each distribution to increase the independence of samples.

When a BBS is used as part of a 3-source capture-recapture method, the 2 unique object distributions should occur before the BBS. This enables individual level data to be more accurately collected in a survey setting by data collectors who receive intensive training rather than by volunteers who may not be able to collect the required data.

## Abbreviations

BBS: biobehavioral survey
CDC: Centers for Disease Control and Prevention
FSW: female sex workers
MOH: Ministry of Health
RDS: respondent-driven sampling
